# Variability of Bacterial Communities in the Moth *Heliothis virescens* Indicates Transient Association with the Host

**DOI:** 10.1371/journal.pone.0154514

**Published:** 2016-05-03

**Authors:** Heike Staudacher, Martin Kaltenpoth, Johannes A. J. Breeuwer, Steph B. J. Menken, David G. Heckel, Astrid T. Groot

**Affiliations:** 1 University of Amsterdam, Science Park 904, 1098 XH, Amsterdam, The Netherlands; 2 Max Planck Institute for Chemical Ecology, Hans-Knöll-Straße 8, 07745, Jena, Germany; Colorado State University, UNITED STATES

## Abstract

Microbes associated with insects can confer a wide range of ecologically relevant benefits to their hosts. Since insect-associated bacteria often increase the nutritive value of their hosts' diets, the study of bacterial communities is especially interesting in species that are important agricultural pests. We investigated the composition of bacterial communities in the noctuid moth *Heliothis virescens* and its variability in relation to developmental stage, diet and population (field and laboratory), using bacterial tag-encoded FLX pyrosequencing of 16S rRNA amplicons. In larvae, bacterial communities differed depending on the food plant on which they had been reared, although the within-group variation between biological replicates was high as well. Moreover, larvae originating from a field or laboratory population did not share any OTUs. Interestingly, *Enterococcus* sp. was found to be the dominant taxon in laboratory-reared larvae, but was completely absent from field larvae, indicating dramatic shifts in microbial community profiles upon cultivation of the moths in the laboratory. Furthermore, microbiota composition varied strongly across developmental stages in individuals of the field population, and we found no evidence for vertical transmission of bacteria from mothers to offspring. Since sample sizes in our study were small due to pooling of samples for sequencing, we cautiously conclude that the high variability in bacterial communities suggests a loose and temporary association of the identified bacteria with *H*. *virescens*.

## Introduction

Symbiotic bacteria of insects provide diverse beneficial services to their hosts, *e*.*g*. upgrading of nutrient-poor diets, digestion of refractory food sources and protection of the host against pathogens [1–4]. Therefore, the study of bacterial communities that are associated with an organism of interest has become an integral part of the fundamental and applied research of, in particular, agricultural pest insects. Investigating bacterial diversity and insect-bacteria associations is greatly facilitated by high-throughput sequencing methods, especially massive parallel amplicon sequencing, that have been developed in the past decade [5–8].

Associations of bacterial communities with a host can be stable or dynamic. Stable associations are characterized by the temporal persistence of bacteria in the host across different life stages and across generations [4,9–12]. The stability of the community can be indicative of the relevance of bacteria for the ecology and evolution of the host. If bacteria are transmitted vertically from one generation to the next for many generations, host and bacteria can be viewed as a unit of selection, and bacteria are often beneficial for the host [9,10,13].

Another criterion to assess the relevance or the functional role of bacteria for an organism is the variability of bacterial communities in the different environments in which the insect host resides [14–18]. Variability of bacterial communities in relation to host diet is of particular interest in agricultural and forest pest insects, because the flexibility of their bacterial communities may enhance the dietary range of phytophagous insects [1,2,4,19–21]. Bacterial communities have been shown to vary depending on diet in several insect species [15,17,22,23] but may also be stable across diets [24]. Other factors that may cause variation in bacterial communities in insects are geographic origin of the host population and rearing history of the insect host [23,25,26].

Studies of bacterial communities in Lepidoptera have accumulated in the past decade, mostly because larvae of many Lepidoptera are major agricultural or forest pests [17,22,23,26,27]. Bacteria from the genus *Enterococcus* have been repeatedly found in Lepidoptera [22,26,28–31] and have been shown to be metabolically active in *Manduca sexta* [29]. Other bacteria that could serve an ecological role in Lepidoptera were isolated from saturnid butterflies and the silkmoth *Bombyx mori* [32,33]. These bacteria exhibited, among others, cellulolytic, pectinolytic and xylanolytic activities that might help caterpillars to digest plant material [32,33]. Furthermore, a recent metagenomic study of the bacterial midgut community in the pyralid moth *Ostrinia nubilalis* revealed the presence of bacterial cellulase-, amylase-, β-galactosidase- and β-glucosidase-encoding genes, indicating the potential of the gut bacterial community to support digestion in its lepidopteran host [23]. However, when the midgut is damaged by insecticidal toxins from spores of *Bacillus thuringiensis*, formerly benign, resident bacteria may breach the midgut barrier, enter the hemocoel and participate in the destruction of the host [34,35].

The noctuid moth *Heliothis virescens* is a major agricultural pest in North and South America [36]. Its larvae are polyphagous and feed on over 37 plant species from 14 families [36–41]. Among these plants are important economic crops such as cotton (*Gossypium hirsutum*), tobacco (*Nicotiana tabacum*) and chickpea (*Cicer arietinum*) [36,37,41,42]. Since bacteria can facilitate host-plant use by herbivorous insects, investigating the bacterial community of *H*. *virescens* and its variation in relation to diet could lead to the identification of bacteria that enhance or inhibit its development and thereby its impact as an agricultural pest.

In this study, we identified and compared bacterial communities in different developmental stages (eggs, larvae and female adults) of *H*. *virescens* from field and laboratory populations that were reared on different plant species (viz. cotton, chickpea and tobacco). Additionally, we investigated the transmission of the bacterial community from one generation to the next.

## Material and Methods

### Ethics statement

*Heliothis virescens* was collected in North Carolina, USA, where this species is a pest and not protected by law. The ARS strain of *H*. *virescens* was collected in 1971 at USDA-ARS property in Washington County (33° 26’ 00” N, 90° 54’ 20” W). Eggs collected in 2011 (see below) were collected at the Oxford Tobacco Research Station in Oxford, North Carolina (36° 18’ 19” N, 78° 36’ 33” W). Eggs were collected by staff of USDA-ARS and North Carolina State University, respectively.

### Effect of host plant species on bacterial community composition of laboratory-reared larvae

To investigate whether the bacterial community composition in *H*. *virescens* larvae changes depending on the food plant species, we used the long-term laboratory-reared ARS strain. This strain was collected from wild hosts in 1971 in Washington County, MS, and since then reared in the laboratory at USDA-ARS in Stoneville, MS. The ARS strain was transferred to the Max Planck Institute for Chemical Ecology (MPI-CE), Jena, Germany in 2010, where the larvae were reared on pinto bean diet containing the antibiotic tetracycline hydrochloride (Sigma-Aldrich, The Netherlands) [43]. Adults were provided with a 10% (v/v) honey water solution. All life stages were kept in climate chambers at a temperature of 25°C, 60% relative humidity and a light/dark cycle of 16h:8h.

We placed 30 first instars of the ARS strain on whole cotton (*Gossypium hirsutum*), chickpea (*Cicer arietinum*) and tobacco plants (*Nicotiana attenuata*), which were all grown in the greenhouse of MPI-CE, Jena, Germany. First instars were randomly picked from offspring of four different mothers. Each plant received larvae from the same four mothers. After nine days, when the larvae were in the second, third or fourth instar, larvae were taken off the plants and starved for eight hours to empty their gut content and to reduce the amount of chloroplasts, as chloroplast DNA may be amplified in the procedure of 16S rRNA amplicon sequencing. To remove bacteria that reside on the outer cuticle, experimental larvae were washed by submerging them for 10 s in 1% (w/v) sodium dodecyl sulfate (SDS) solution and for 10 s in sterile water. Larvae were then put in 1.5-ml tubes and immediately placed on ice, to prevent changes in the bacterial community composition, after which the samples were stored at -80°C [44]. For experimental design, see also the flowchart (S1 Fig).

### Effect of host plant species on bacterial community composition of field larvae

To investigate bacterial communities of a field population of *Heliothis virescens*, eggs of this moth were collected in July 2011 in North Carolina, USA, on commercially grown tobacco, *N*. *tabacum*. These eggs were allowed to hatch and the resulting larvae (referred to as field larvae), adult females as well as the eggs laid by these females were directly used to assess bacterial communities. The field-collected eggs were divided into three groups in plastic cups (100 ml) which were filled with leaf material of one of the three plant species cotton (*G*. *hirsutum*), chickpea (*C*. *arietinum*) and tobacco (*N*. *tabacum*). After emergence, first instars were transferred to whole plants. We used ten plants per host plant species (thus 30 plants in total), and each plant received 20–30 first instars. The plants were kept outdoors until experiments started (*i*.*e*. when larvae were placed on them to feed). Each plant was kept in an individual cage made of fine-meshed gauze (diameter: 60 cm, height: 1 m). After larvae had reached the fifth instar, one larva was randomly collected from each plant batch and starved for eight hours. These 30 larvae were washed by submerging them for 10 s in 1% (w/v) SDS solution and for 10 s in sterile water. After the wash step, guts were extracted by cutting the larva between the 1^st^ and 2^nd^ thoracic and between the 7^th^ and 8^th^ abdominal segments. Dissected guts were stored at -80°C until DNA extraction. The remaining larvae were left to pupate in the soil of the potted whole plant and subsequently used in the bacterial transmission experiment (see below). For experimental design see also flowchart of S1 Fig.

### Diversity and composition of the bacterial community in adult females and their eggs

To determine bacterial communities in females and their corresponding eggs, a subset of the field larvae that was not used to determine the larval bacterial community, was allowed to pupate in the soil and develop into adults. Upon emergence, males and females that had fed on the same plant species were mated in carton cups (200 ml) in single pairs and provided with cotton that was soaked with 10% (w/v) sugar water solution. The cups also contained leaves of the same plant species on which the mating pairs had been as larvae to stimulate, and provide a substrate for, oviposition. After four days, we collected abdomens from three females and 30 of their eggs per plant species group, and thus obtained nine female-egg combinations in total. DNA from eggs was extracted in batches of 30 eggs per female. Eggs were collected from the plant leaves with a brush, transferred to 1.5-ml tubes and placed immediately on ice until storage at -80°C. Female abdomens were cut off and submerged in 1% (w/v) SDS solution for 10 s, followed by a 10 s washing step in sterile water. Abdomens were then transferred to 1.5-ml tubes and placed immediately on ice until storage at -80°C. All instruments were rinsed with 1% (w/v) SDS solution and sterile water between samples. All tissues were collected in 1.5-ml tubes and placed immediately on ice and stored at -80°C.

### Effect of laboratory rearing and antibiotics treatment on bacterial communities of field larvae

To assess whether bacterial communities change after transfer from the field to the laboratory and how the antibiotic tetracycline, which is normally part of the laboratory diet (see above), affects the bacterial gut community, moths that originated from eggs that were collected in the field in 2011 to assess bacterial communities (see above) were kept in the laboratory at the University of Amsterdam for three generations (in the following referred to as field-lab larvae). Larvae were kept on artificial pinto-bean diet, but without the addition of antibiotics. Females of the third generation were mated in single pairs in empty 200-ml plastic cups. Eggs of the fourth generation hatched in these cups, and the resulting first instars were transferred to cotton leaves with (AB group) or without tetracycline coating (NoAB group). Every other day, cotton leaves were coated with tetracycline by pipetting 1 ml 0.1% (w/v) aqueous tetracycline solution onto the upper leaf surface. Control leaves were treated in the same way with only water. In addition, we wrapped the leaf stems with cotton soaked with 0.1% (w/v) aqueous tetracycline solution in the AB group or water in the NoAB group, respectively. When the larvae reached the fifth instar, they were starved for eight hours, then washed by submerging them for 10 s in 1% (w/v) SDS aqueous solution and for 10 s in sterile water, after which their gut was extracted as described above. The guts were stored at -80°C until DNA extraction.

### DNA extraction

DNA from the field and field-lab population was extracted by grinding samples in 500 μl TES buffer [100 mM tris(hydroxymethyl) aminomethane hydrochloride (TRIS-HCl) pH 8, 10 M ethylenediaminetetraacetic acid (EDTA), 2% SDS] and 4 μl lysozyme from chicken egg white (100 mg/ml) (Sigma-Aldrich), after which the samples were incubated at 37°C for 30 min. Next we added 2.5 μl proteinase K (20 mg/ml) (Sigma-Aldrich) and incubated the samples for 56°C overnight. The rest of the extraction procedure was conducted according to the standard CTAB/chloroform protocol as described in Unbehend et al. (2013) [45]. DNA from the lab-strain larvae was extracted with the Epicenter MasterPure kit (Epicenter Technologies, USA) according to manufacturer’s instructions. Additionally, a lysozyme step [4 μl lysozyme (100 mg/ml), incubated at 37°C for 30 min] was added before the proteinase K step.

### Bacterial tag-encoded FLX amplicon pyrosequencing (bTEFAP)

Samples were pooled for sequencing as depicted in S1 Fig. For the larvae of the laboratory strain, we made three pools, one pool contained DNA of seven larvae that were reared on each of the three plant species (cotton, tobacco or chickpea). For the larvae of the field population, we made six pools of DNA, two pools (biological replicates) for each plant species, to assess the variation between biological replicates; each pool contained the DNA of five larvae. For the field-lab population, we made two pools, one pool of 45 NoAB larvae and one pool of 49 AB larvae. Before pooling, individual samples were diluted to 25 ng/μl and added in equal volumes to one pool. Pooled samples were sent to an external service provider (Molecular Research Lab, MR DNA, Shallowater, TX, USA) for bTEFAP, using the 16S rRNA primers Gray28F (5’-GAGTTTGATCNTGGCTCA-3’) and Gray519R (5’-GTNTTACNGCGGCKGCTG-3’) [46]. A sequencing library was constructed via one-step PCR with 30 cycles, using a mixture of Hot Start and HotStar hi-fidelity polymerases (Qiagen). Sequencing was performed on a Roche 454 FLX instrument with Titanium reagents and procedures and protocols of Molecular Research LP (http://www.mrdnalab.com/).

### Sequence analysis

Analysis of the sequences was performed in QIIME (quantitative insights into microbial ecology), which is a standard pipeline to analyze microbial communities based on 16S rRNA amplicon sequencing [47]. Raw data were de-noised with denoise_wrapper [48]. Low-quality reads (quality cut-off = 25) and sequences that were shorter than 200 bp or longer than 600 bp were excluded from further analysis. The remaining sequences were clustered into operational taxonomic units (OTUs) with the open-reference OTU picking command, using uclust (version 1.2.22) [49] and applying 97% similarity cut-offs. Sequences were first clustered against a reference data set (http://greengenes.lbl.gov/). The sequences that did not cluster with the reference sequences were clustered de novo and checked for chimeras with Chimera Slayer [50]. Identified chimeras were removed from the dataset for downstream analysis.

From each OTU cluster, the most abundant sequence was taken as the representative sequence. Taxonomy was assigned to the representative sequences with the uclust consensus taxonomy classifier. The resulting OTU table was manually edited, and global singletons, chloroplasts and mitochondria were removed (see S1 Table for number of chloroplast and mitochondrial sequences in the dataset). Representative sequences of the remaining OTUs were aligned with PyNAST, using the Greengenes core set (http://greengenes.lbl.gov/) as a template [51]. A phylogenetic tree was constructed with the open source software Fasttree 2.1.3, applying the generalized time-reversible (GTR) model of nucleotide evolution [52]. Local support values for tree splits were calculated with the Shimodaira-Hasegawa test [53]. This phylogenetic tree, which included all identified OTUs, was used to calculate the weighted UniFrac metric [54] which was used as the basis for principal coordinates analysis (PCoA) (see below).

### Diversity and phylogenetic analysis

Diversity analysis of our samples was done with the QIIME pipeline. Since our samples showed unequal sampling depth (see S2–S4 Tables), we investigated alpha- and beta-diversity of the bacterial communities of our samples with rarefied OTU tables. The number of sequences used for rarefaction corresponded to the number of sequences present in the sample with the lowest number of sequences (339 sequences). To assess alpha-diversity, we calculated the Shannon index based on the rarefaction tables [55]. To identify possible clusters based on similarity of bacterial communities among our samples, we produced a PCoA plot on the basis of weighted non-normalized UniFrac distances and with rarefied OTU tables [54]. To assess the robustness of our results, we additionally performed a Jackknife analysis on weighted UniFrac distances, which is included in the QIIME pipeline [47,54,56]. Jackknife support is included in the PCoA plots as the size of the ellipsoid areas surrounding the data points.

### OTU patterns across samples

**Fig 1:**
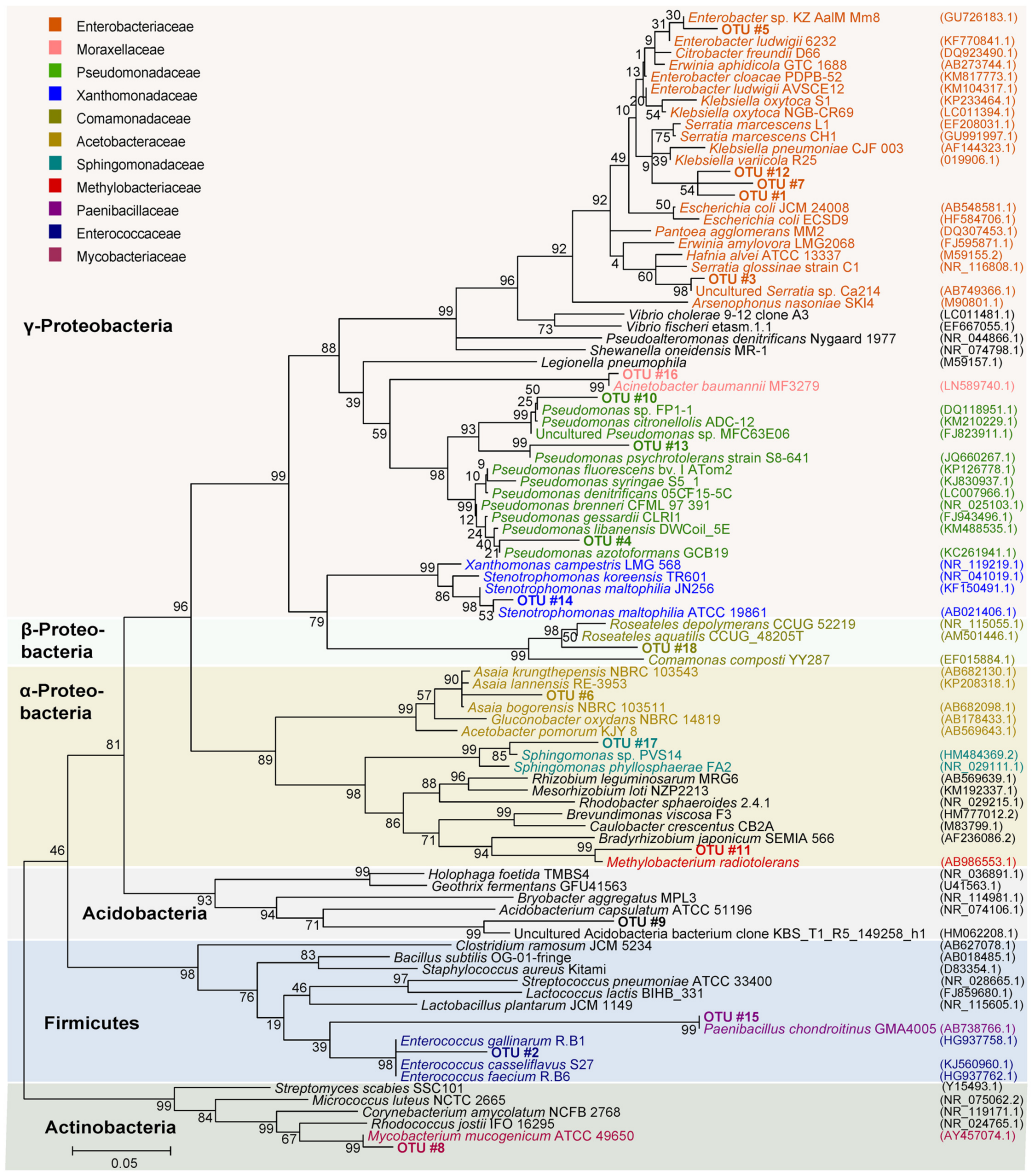
Maximum likelihood phylogenetic tree of bacterial OTUs with an average presence of more than 1% across all samples. OTUs that were identified in *H*. *virescens* are indicated in bold font and labelled OTU #1 to OTU #18. Reference sequences were obtained from Genbank; strain names are given after the species or genus name. Accession numbers are between brackets. Font colors correspond to different bacterial families, background colors indicate bacterial phyla (or class in the Proteobacteria). The tree was constructed on the basis of partial 16S rRNA gene sequences, applying 500 bootstrap replications. Bootstrap values are indicated above the branches.

None of the bacterial OTUs was present in all samples, *i*.*e*. present in samples of all origins, rearing plants, and life stages. Most common were three OTUs, which were present in 65% of the samples. These OTUs were mainly present in females and eggs, which had a high sample size compared to the other groups. To also consider OTUs that were present in the larvae, we filtered for OTUs that were on average represented more than 1% across all samples (*i*.*e*. first the percentage of an OTU in a sample was calculated followed by the average of the percentage of the OTU in all samples, see Table 1), resulting in 18 OTUs. To classify and construct a phylogenetic tree of these 18 OTUs, we first aligned the sequences of our dataset and 16S rRNA reference sequences obtained from Genbank [57], using ClustalW [58], after which we constructed a maximum likelihood tree in MEGA 6 [59] with the Tamura-Nei model [60], using uniform rate variation and 500 bootstrap replicates. To root the tree, we used three Archaea as outgroups: *Halobacterium salinarum* (NR_113428.1), *Sulfolobus acidocaldarius* (NR_074267.1) and *Pyrolobus fumarii* (NR_102985.1). Outgroup taxa were excluded from the figure for clarity (Fig 1).

**Table 1:** Bacterial OTUs with an average presence of more than 1% across all samples.

#	Family (Genus)	Total Average (29)	Larvae Lab (3)	Larvae Field (6)	Larvae Field-lab (2)	Adults (9)	Eggs (9)	Overall Presence > 1%
**1**	Enterobacteriaceae (*Klebsiella/Enterobacter*)	**27.63**	0.00	0.00	35.62	72.34	35.95	68.97
**2**	Enterococcaceae (*Enterococcus*)	**11.40**	50.55	0.00	61.70	0.00	0.01	17.24
**3**	Enterobacteriaceae (*Serratia*)	**8.57**	0.00	0.00	0.01	7.20	22.42	44.83
**4**	Pseudomonadaceae (*Pseudomonas*)	**4.82**	0.00	0.00	0.77	0.69	19.62	55.17
**5**	Enterobacteriaceae (*Enterobacter*)	**3.97**	0.00	0.00	0.01	5.06	5.29	20.69
**6**	Acetobacteraceae (*Asaia*)	**2.38**	49.45	0.00	0.00	0.00	0.00	6.90
**7**	Enterobacteriaceae (unclassified)	**2.15**	0.00	7.75	0.00	0.00	0.00	6.90
**8**	Mycobacteriaceae (*Mycobacterium*)	**2.15**	0.00	0.00	0.00	0.03	4.13	27.59
**9**	Acidobacteria (unclassified)	**2.04**	0.00	8.42	0.00	0.00	0.00	3.45
**10**	Pseudomonadaceae (*Pseudomonas*)	**1.87**	0.00	0.00	0.04	5.32	0.02	3.45
**11**	Methylobacteriaceae (*Methylobacterium*)	**1.57**	0.00	31.45	0.00	0.00	0.00	13.79
**12**	Enterobacteriaceae (*Klebsiella/Enterobacter*)	**1.57**	0.00	0.00	0.02	1.92	2.46	13.79
**13**	Pseudomonadaceae (*Pseudomonas*)	**1.54**	0.00	0.00	0.00	0.17	2.55	20.69
**14**	Xanthomonadaceae (*Stenotrophomonas*)	**1.39**	0.00	0.00	1.55	4.59	0.20	34.48
**15**	Paenibacillaceae (*Paenibacillus*)	**1.12**	0.00	18.19	0.00	0.00	0.00	13.79
**16**	Moraxellaceae (*Acinetobacter*)	**1.04**	0.00	0.00	0.04	0.08	6.53	24.14
**17**	Sphingomonadaceae (*Sphingomonas*)	**1.02**	0.00	0.00	0.01	0.00	0.16	6.90
**18**	Comamonadaceae (*Roseateles*)	**1.00**	0.00	8.84	0.07	0.00	0.01	13.79

Taxonomy and percentage of relative abundance in different developmental stages (egg, larva and adult) and populations (laboratory, field and field-lab). Sample sizes are given between brackets.

#: OTU identification number

### Statistical analysis of similarities between bacterial communities of females and their eggs

Stability of bacterial communities from one generation to the next was assessed by testing whether egg bacterial communities resemble the bacterial community of their mother with a linear mixed model in the package lme4 [61] and lmerTest [62] in the software R, version 3.0.2 [63]. Weighted UniFrac distance values of all 81 female-egg combinations were used as response variable. UniFrac distance values were calculated based on rarefied OTU tables, which were also used to produce the PCoA plots (see above). We used two predictor variables: female-egg combination (eggs belonged to one female or not) and plant species (eggs belonged to the same plant species—cotton, chickpea, or tobacco—as a female or not). To account for repeatedly entering females and eggs into the model, we added egg and female as random factors to the model. Because there was no interaction effect between plant and female-egg combination, we excluded the interaction term from the model. Degrees of freedom were approximated using the Satterthwaite method. As a *post hoc* test for the plant effect, we performed planned pairwise comparisons between female-eggs combinations of one plant species group and female-egg combinations in which females did, but eggs did not belong to that respective plant group, by using the multcomp package [64] in R, applying a Bonferroni correction for multiple comparisons.

### Availability of supporting data

The data sets supporting the results of this article are available at the Dryad Digital Repository, doi:10.5061/dryad.dv35j.

## Results

### Bacterial community composition of *H*. *virescens*

We received a total of 312,324 sequences, of which 295,289 sequences remained after quality filtering. Other sequences were global singletons or chimeras, chloroplasts and mitochondria (also see S1 Table). The bacterial sequences clustered into 566 OTUs, of which 42 were unassigned. An overview of numbers of sequences before and after quality filtering, as well as numbers of OTUs in the different samples is given in S2–S4 Tables.

None of the OTUs in *H*. *virescens* was present in all samples (n = 29), and only three OTUs were found in more than 65% of our samples (65% was the highest percentage of samples in which OTUs were commonly present). When we assessed how many OTUs had an average percentage of more than 1% across all samples, this resulted in 18 OTUs, but only two of these were present with an average percentage of more than 10% across all samples (Table 1; see Fig 1 for phylogenetic position).

### Variability of bacterial communities between laboratory and field larvae

**Fig 2:**
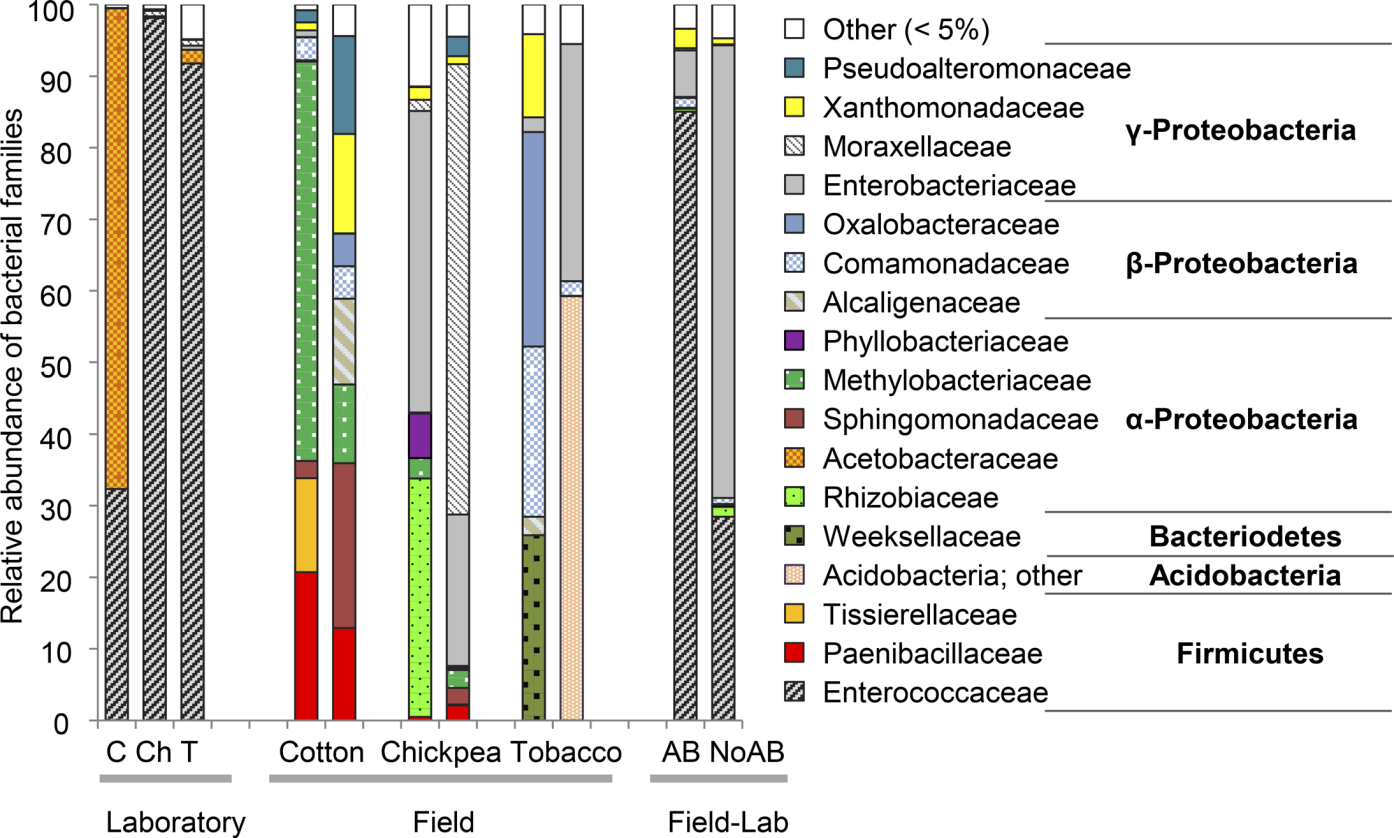
Relative abundance of bacterial OTUs in *H*. *virescens* larvae. Bacterial OTUs were combined on the family level. Families that were not represented with a minimum of 5% in at least one of the samples are combined under “other”. Larvae originated from a laboratory colony, from the field or from the field, after which they were reared in the laboratory for four generations (field-lab): AB: larvae received antibiotics treatment, NoAB: larvae received no antibiotics treatment. Field and laboratory larvae were grown on cotton (C), chickpea (Ch) or tobacco (T).

We compared bacterial communities between larvae from a long-term laboratory colony and larvae that had been collected in the field as eggs. Laboratory and field larvae had no OTUs in common. The bacterial community of lab larvae was dominated by two OTUs that were classified as *Enterococcus* sp. (Lactobacillales) (#2) and *Asaia* sp. (Rhodospirillales) (#6), whereas larvae of the field population did not contain any OTUs affiliated with these two genera. In fact, field larvae did not contain clearly dominant OTUs, but harbored Enterobacteriales, Burkholderiales or Rhizobiales in relatively high percentages (see below). Furthermore, bacterial communities in the field-lab larvae differed greatly from the bacterial communities of the field larvae: only 1.4% of the OTUs (n = 148) of the field larvae were detected in the field-lab larvae that were reared without antibiotics (NoAB). *Enterococcus* sp. (#2) was more abundant in the AB field-lab larvae than in NoAB larvae (Fig 2).

**Fig 3:**
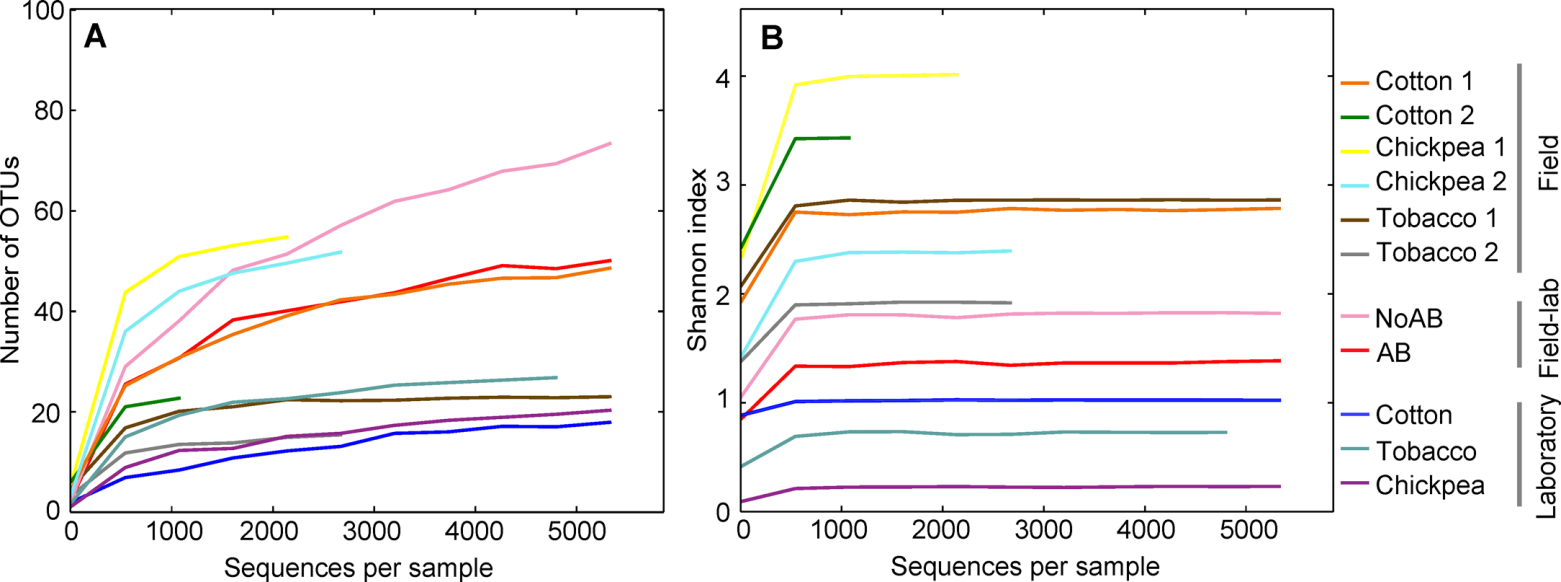
Rarefaction curves of alpha diversity of the bacterial communities of *H*. *virescens* larvae. (**A)** Number of observed bacterial OTUs. (**B)** Shannon index of the bacterial communities. Larvae came from a laboratory colony, were collected from the field as eggs, or were collected from the field as eggs and were reared in the laboratory for four generations (field-lab). AB: field-lab larvae that received antibiotics during one generation; NoAB: field-lab larvae that did not receive antibiotics treatment.

When we investigated the alpha-diversity of the larvae, larvae from the laboratory colony harbored an average of 25 OTUs, while field larvae had an average of 35 OTUs. Additionally, communities of the field larvae showed a greater evenness than those of the laboratory larvae (Fig 2), which was reflected in the higher Shannon index of the field larvae (Fig 3).

### Host plant-associated variability of bacterial communities in larvae

The influence of diet on the bacterial communities of laboratory and field larvae was investigated by comparing bacterial communities in larvae that fed on three different host plant species. In laboratory-reared larvae, we found one OTU (#2) from the family Enterococcaceae dominating the bacterial community in the guts of larvae fed chickpea and tobacco, *i*.*e*. 97.8% and 91.5% abundance, respectively (Fig 2). The closest hits for OTU #2 were *Enterococcus faecium* (HG937762), *E*. *gallinarum* (HG937758) and *E*. *casseliflavus* (KJ528946) with 99% similarity. The larvae fed on cotton also contained OTU #2, but in much lower abundance, *i*.*e*. 31.8%, while an OTU of the family Acetobacteraceae (#6) had the highest abundance (67.1%). The closest hit for this OTU was *Asaia lannensis* (KP208318), with 99% similarity.

In the field larvae, we also found differences in bacterial communities depending on the host plant species they fed on. Enterobacteriaceae were the dominant family in the larvae that fed on chickpea and also occurred in larvae that fed on tobacco, while these were rare (< 1%) in the larvae fed on cotton. The larvae fed on tobacco and cotton contained Burkholderiales (Comamonadaceae, *e*.*g*. #18, and Oxalobacteraceae), which were rare (< 1%) in larvae fed on chickpea (Fig 2). Methylobacteriaceae (*e*.*g*. #11) were among the most abundant families in the larvae that fed on cotton, while this family was hardly detected in the other two groups. On the genus (and OTU) level, the two biological replicates from each plant group differed greatly from each other (S2 Fig).

### Variability of bacterial communities across different life stages of the field population

**Fig 4:**
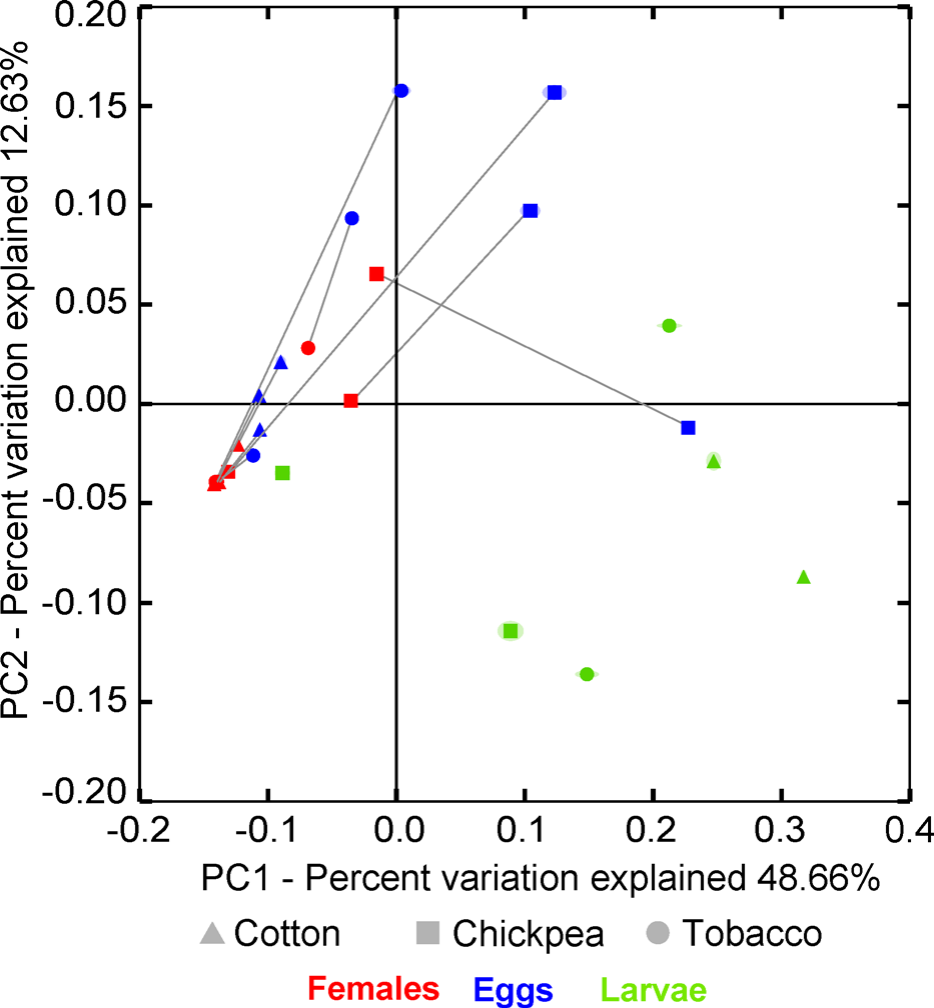
Principal coordinates analysis plot of bacterial communities of field larvae, female adults and their eggs. Absence of confidence ellipsoids around the samples indicates good jackknife support for the location of data points in the PCoA plots. Colors indicate life stages; symbols denote food plant; lines connect female-egg combinations.

We assessed the temporal persistence of bacterial communities in *H*. *virescens* by comparing bacterial communities of different life stages (larvae, adults and eggs) of the field population. Bacterial communities differed greatly across life stages. Females shared 76% of their OTUs (n = 200) with eggs, and eggs shared 52.1% of their OTUs (n = 292) with adult females. In contrast, larvae only shared 2.7% of their OTUs (n = 148) with females and 6.1% OTUs with eggs. Accordingly, in the PCoA plot, females and eggs partly clustered together, while five of the six larval samples were located in a different area of the plot (Fig 4). Females of the field population shared 22% of their OTUs (n = 200) with the field-lab larvae that were reared in the laboratory for four generations (the NoAB group), while the eggs had 18.8% of their OTUs (n = 292) in common with the field-lab larvae.

One OTU (#1) from the family Enterobacteriaceae had the highest relative abundance in both females and eggs (S3 Fig). The closest hits for this OTU in NCBI were *Enterobacter ludwigii* (KJ767368), *E*. *cloacae* (KM817773) and *Klebsiella oxytoca* (KM408615), all with 97% similarity; this was confirmed by its position in the phylogeny (Fig 1). In the field larvae, OTU #1 was not present, but two other Enterobacteriaceae OTUs were found (#7 and #12), which clustered with OTU #1 in the phylogenetic tree (Figs 1 and 2).

### Vertical transmission of bacterial communities

Based on weighted UniFrac distance values, bacterial communities present in females were not more similar to the bacterial communities of their own eggs than to the bacterial communities of eggs that were not their own (F_1,65.74_ = 1.28, P = 0.26). However, rearing on different plant species had a significant effect on bacterial communities in female-egg combinations (F_5,66.53_ = 3.17, P = 0.013). Yet, none of the comparisons for the variable plant species were significant in planned pairwise comparisons with Bonferroni corrections (see also the PCoA plot, Fig 4 and S3 Fig).

## Discussion

In this study we assessed the variability of bacterial communities in the agricultural pest, the moth *H*. *virescens*. We observed high variability of bacterial communities among different life stages, different populations (field population or laboratory colony) as well as of larvae that fed on different host plant species, and even of biological replicates.

### Abundant bacterial strains in *H*. *virescens*

Most of the dominant bacterial families in our study, *i*.*e*. Acetobacteraceae, Methylobacteriaceae, Sphingomonadaceae, Comamonadaceae, Enterobacteriaceae, Pseudomonadaceae, Moraxellaceae Xanthomonadaceae, Paenibacillaceae and Enterococcaceae, have been identified in Lepidoptera before [14,17,22,23,26,30,31,65]. This suggests that representatives of these bacterial families may be commonly present in many Lepidoptera and the environments they live in.

### Variability of bacterial communities between laboratory and field larvae

Bacterial communities differed greatly between laboratory and field larvae, in fact no OTUs were shared between the two groups. These large differences in bacterial communities underline the importance of including field populations of an organism in studies of the functional role of its bacterial communities. Bacterial communities of field and laboratory insect populations have for instance been studied in the moths *Helicoverpa armigera* [26] and *Ostrinia nubilalis* [23], the mosquito *Anopheles stephensi* [66] and several fruitfly species (*Drosophila*) [15]. In all studies, the general finding was that bacterial communities in laboratory reared insects are depauperate and dominated by one (or no more than a few) bacterial strain(s), as we also found in *H*. *virescens*, while communities of field populations tend to be much more diverse and contain many bacterial strains with lower and more equal abundances [15,23,26,66]. Differences between laboratory and field populations may be due to differences in environmental conditions. In the field, organisms are probably exposed to a higher diversity of bacterial strains which may vary in space and time, than in the laboratory. For instance, in *H*. *virescens*, as in most moth species, eggs are laid on above-ground parts of plants, where larvae also feed, while the moths pupate in the soil. Emerging adults feed from flowers of many plants and, in the case of the polyphagous *H*. *virescens*, many plant species [36–39]. In contrast, in the laboratory moths are reared in cups on artificial diet, where they also pupate and emerge as adults. Thus, whereas moths in the field may continuously encounter and ingest new strains of bacteria, lab colonies are likely constantly re-infected with the same, small number of bacterial strains that are dominant under laboratory conditions. Importantly, the field larvae of our study have been kept in gauze cages in the greenhouse from the egg stage onwards. We chose this approach because when we collected *H*. *virescens* larvae from the field in previous years, a high percentage of the larvae was parasitized by wasps (unpublished data) which might affect bacterial community composition. Possibly, larvae that are sampled directly from the field would exhibit an even more diverse bacterial community than what we found, with greater differences compared to the laboratory larvae. Moreover, it should be noted, that our results are based on three pools of laboratory larvae and six pools of field larvae. To firmly test for differences in alpha diversity between field and laboratory larvae in *H*. *virescens*, larger samples sizes are necessary.

As described in the methods, we used whole (washed) larvae to assess the bacterial communities in the laboratory strain and extracted the guts of larvae to assess bacterial communities in field larvae. Also, laboratory strain larvae were in an earlier instar than field larvae. Even though the differences in treatment and instar might be responsible for part of the differences between field and laboratory larvae in our study, they are unlikely to explain the large qualitative differences with a complete lack of overlap in bacterial communities. This is particularly true of the finding that *Enterococcus* sp. which was absent in field larvae but the dominant strain in laboratory larvae, was also detected in the field-lab larvae that were reared in the laboratory for four generations. These field-lab larvae were processed exactly like the field larvae (gut extraction of fifth instar larvae and DNA extraction protocol), which makes it unlikely that the presence of enterococci is due to methodological differences between laboratory-reared and field larvae.

All larvae that were used for bacterial community analysis were starved for eight hours. This was done to let the larvae excrete plant material and thus to reduce the amount of chloroplast in the samples, as chloroplasts also contain the 16S rRNA gene which can be amplified in a 16S rRNA gene amplicon study like ours. However, starvation can induce hormonal and metabolic changes, as has been found in caterpillars of the moth *Manduca sexta* [67,68]. Such starvation-induced changes may also affect bacterial community composition, which should be investigated in future studies.

In our study, *Enterococcus* sp. was the dominant bacterial strain in the laboratory colony and the antibiotics treated (AB) group of the field-lab larvae, and it was the second most abundant strain in the not antibiotics treated (NoAB) group of the field-lab larvae. However, this bacterium was completely absent in field larvae, suggesting that *Enterococcus* sp. was introduced to the insects in the laboratory. *Enterococcus* sp. has been reported as a dominant bacterium in several Lepidopteran gut communities, including *Galleria mellonella*, *Lymantria dispar*, *Helicoverpa armigera*, *Manduca sexta*, *Spodoptera littoralis* and *Heliconius erato* [22,26,28–31]. Interestingly, most of these studies used only laboratory colonies [28–30]. In *Helicoverpa armigera* and *Ostrinia nubilalis*, where both laboratory and field populations were investigated [23,26], single bacterial strains such as *Enterococcus* sp. and other gram-positive cocci (*e*.*g*. *Staphylococcus* sp. in *O*. *nubilalis*) were found to become dominant in laboratory populations, while they were less abundant in field populations. In *H*. *erato*, enterococci became more abundant in adults after this species had been in captivity for one generation (from adult to adult) compared to field-collected adults [31]. Possibly, enterococci are outcompeted in the field by other bacteria (*e*.*g*. Proteobacteria such as *Enterobacter*, *Klebsiella* and *Pseudomonas* species) in the insect gut and/ or on the plants while competition changes in favor of enterococci under (more stable) laboratory conditions. One possibility that we cannot completely rule out is that *Enterococcus* was present in the field larvae in very low numbers, but was not amplified during sequencing. However, this is not likely because of the high sequencing depth that we used. *Enterococcus* was present in some of the egg samples (see Table 1) in very low numbers (one, two or three reads). Since there were no enterococci reads in the field larvae, we suspect that *Enterococcus* was introduced to adults and eggs (possibly in the paper cups in which adults were mated and eggs were laid).

Treating *H*. *virescens* larvae with tetracycline in the laboratory possibly selects for enterococci. Many *Enterococcus* strains are known to have developed resistance against tetracycline, especially in the context of animal agriculture [69–71]. To our knowledge, there are no reports of tetracycline resistance of enterococci in *H*. *virescens* or its food plants. In our study, enterococci were abundant in the laboratory strain that has been on antibiotics for many years before our study. However, enterococci were highly abundant in both the antibiotics and non-antibiotics groups of the field-lab larvae, indicating that antibiotic treatment alone does not account for the shift in microbial communities towards high abundance of *Enterococcus* species.

### Host plant-associated variability of bacterial communities in larvae

In both field and laboratory *H*. *virescens* larvae, bacterial gut communities varied depending on host plant. Diet has been found to influence bacterial gut community composition in larvae of many insect species, *e*.*g*. *Drosophila* [15] and several lepidopteran species [17,22,30]. Diet might influence bacterial communities in various ways. For example, different bacteria may be present in or on different host plants, the resources provided by the different plants may promote differential bacterial growth and/or secondary plant metabolites may have a selective effect on bacterial communities [72,73]. Additionally, host plants can influence the physiochemical conditions of the larval gut, which possibly results in differential bacterial growth [74,75].

Although we did not sample bacteria from plants on which the larvae had fed, earlier studies have assessed bacterial communities of cotton and tobacco plants, and identified several bacterial families that we also detected in our samples [29,76], suggesting that the experimental *H*. *virescens* larvae had ingested bacteria from their food plants. For instance, Methylobacteriaceae was the most abundant bacterial family in the field larvae fed on cotton in our study, and this bacterial family has been isolated from different tissues of cotton [76,77]. Also, bacteria from the order Burkholderiales (Comamonadaceae and Oxalobacteraceae), which were mainly present in the field larvae fed on tobacco in our study, were previously isolated from tobacco leaves [29]. Furthermore, *Enterobacter*, *Pseudomonas* and *Serratia* were the most abundant genera in females and eggs in our study, and have been isolated from, among others, cotton tissues and tobacco leaves [76,78–80].

There were notable differences in bacterial community composition between the two biological replicates per plant species in the field larvae, particularly at low taxonomic levels (*i*.*e*. genus or OTU). This indicates large individual variation in the bacterial gut community of *H*. *virescens* larvae in the field, and further suggests that the bacterial community is only loosely associated with this moth. As we pooled samples in our study and our sample sizes were small, future studies should investigate the bacterial community of individual larvae in both laboratory and field larvae with a samples size that allows statistical testing for an effect of host plant and to assess the variability between individuals.

When investigating microbial communities using bacterial tag-encoded FLX pyrosequencing of 16S rRNA amplicons, it is important to keep in mind that this method can lead to incorrect estimates of OTU numbers [81–83]. Errors that have been described for this method include PCR bias, sequencing errors (particularly in homopolymeric regions) and the production of chimeric sequences during PCR reactions [5,50,82,84]. Even though we have implemented in our analyses a number of measures to decrease methodological errors (de-noising raw data, quality cut-off, length limitation of sequences, identification and removal of possible chimeric sequences, removal of global singletons), it is possible that the number of OTUs may still be overestimated and that the diversity patterns that we show on the family level for larvae are actually more realistic than the patterns we encountered on the genus (or OTU) level (compare Fig 2 and S2 Fig).

### Variability of bacterial communities across different life stages of the field population

In *H*. *virescens*, it seems that bacterial communities are completely restructured or lost during metamorphosis from crawling larvae to winged adults, because bacterial communities in larvae of the field population differed strongly from those in adult females. One of the few Lepidoptera where bacterial persistence across metamorphosis has been studied is the butterfly *H*. *erato* [31]. In this species, bacterial communities between larvae and adults also differed greatly but more bacterial strains (13%) overlapped between larvae and adults in their study than in ours, in which only 2.7% of OTUs overlapped between larvae and adult females. A similar pattern was found in mosquitoes, in which bacterial composition partly changed during metamorphosis, but many strains were retained [66]. One reason for the difference in bacterial communities between larvae and adults in Lepidoptera might be that larvae and adults feed on different diets: while larvae usually feed on the foliage of their host plants, adults often feed only on nectar, and in this study on honey water. The gut of lepidopteran larvae is less compartmentalized then the gut of many other insect species, mostly has a very alkaline pH, and is purged before metamorphosis [4,75,85–87]. Together, these characteristics may make it difficult for bacteria to successfully colonize Lepidopteran guts. Moreover, in moths an increase in lysozyme production before and during metamorphosis has been reported, which likely reduces bacterial abundance and diversity, especially immediately before and after the pupal stage, *i*.*e*. pupating larvae and newly eclosed adults [88,89]. Upon adult eclosion, bacterial titers may therefore be low and bacterial strains that are different from the larval bacterial community might be able to colonize the adults (see also [31]). However, a recent study on the association between *Enterococcus mundtii* and its lepidopteran host *G*. *mellonella* showed that the symbiont was retained in the host during metamorphosis. Moreover, *G*. *mellonella* and *E*. *mundtii* seem to hamper bacterial species, such as *Serratia* sp. and *Staphylococcus* sp. from colonizing the moth or from being retained until the adult stage [90]. When lysozyme production was impeded in *G*. *mellonella* via RNAi knock-down, *Serratia* sp. significantly increased in number compared to wild type hosts, suggesting that lysozyme production in *G*. *mellonella* suppresses *Serratia* sp., but not *E*. *mundtii*. Further, *Serratia* sp. and *Staphylococcus* sp. became more abundant than *E*. *mundtii* when the latter did not produce bacteriocin, suggesting that bacterial competition in the host plays a major role in shaping the bacterial community [90].

### Vertical transmission of bacterial communities

We investigated whether bacteria in *H*. *virescens* are vertically transmitted by comparing bacterial communities of females and their eggs and tested if bacterial communities of eggs were more similar to bacterial communities of their own mother than to those of other females. The fact that we did not find this effect suggests that vertical transmission likely does not occur in *H*. *virescens* at a significant rate. However, since bacterial communities were at least partly similar in females and eggs, we cannot completely exclude the occurrence of vertical transmission. A study using fluorescently-labelled *E*. *coli* was able to detect a certain amount of mother-offspring transmission in *G*. *mellonella*, although viability of the bacteria was not assessed [91]. Also, a previous study about vertical transmission of *Serratia marcescens* in a laboratory population of *H*. *virescens* indicated that bacteria can be vertically transmitted in this moth [92].

## Conclusions

The huge variability of bacterial communities that we found between life stages, diets, biological replicates and field and lab populations indicates that the major part of the bacterial communities that we identified in the gut of *H*. *virescens* is of a transient nature and only loosely associated with its host. In fact, the bacterial communities seem to be entirely restructured during metamorphosis. Our results further suggest that bacterial communities are not transmitted at a significant rate from mothers to eggs in *H*. *virescens*. Based on these results, it is doubtful that particular bacterial strains that we have identified form a unit of selection with its host. It is further unlikely that a long-term mutualistic symbiosis between *H*. *virescens* and bacteria that could facilitate host plant use and adaptation has evolved. Importantly, our data suggests that enterococci might have been introduced to *H*. *virescens* larvae in the laboratory. This finding stresses the importance of including field populations when bacterial communities of an organism are to be characterized.

## Supporting Information

S1 Fig Schematic overview of experimental design and sample pooling. (TIF)

S2 Fig Relative abundance of bacterial genera in *H*. *virescens* larvae. Rare OTUs that were not represented more than 0.5% in at least one sample are not included. Larvae originated from a laboratory colony, from the field or were collected from the field as eggs, after which they were reared in the laboratory for four generations (field-lab): AB: larvae received antibiotics treatment, NoAB: larvae received no antibiotics treatment. Field and laboratory larvae were grown on cotton (C), chickpea (Ch) or tobacco (T). Brackets around genera signify that there were bacterial candidates with > 97% identity, but support in the phylogenetic tree was weak [*i*.*e*. nodes that divide branches that contain different genera have bootstrap values equal to or below 10 (see Fig 1)].
(TIF)

S3 Fig Relative abundance of bacterial genera in the abdomen of *H*. *virescens* females (F) and their eggs (E). Females (F1-F9) and their eggs (E1-E9) are shown next to each other. Females had fed as larvae on three different plants: cotton (C), chickpea (Ch), tobacco (T). Rare OTUs that were not represented more than 0.5% in at least one sample are not included. Brackets around genera signify that there were bacterial candidates with > 97% identity, but support in the phylogenetic tree was weak [*i*.*e*. nodes that divide branches that contain different genera have bootstrap values equal to or below 10 (see Fig 1)].
(TIF)

S1 Table Percentage of chloroplast and mitochondrial reads in the different sample groups. Laboratory larvae, larvae that were collected in the field as eggs (field), larvae that were collected in the field as eggs and were kept in the laboratory for four generations (field-lab), female adults and eggs.
(DOCX)

S2 Table Statistics of bacterial tag-encoded FLX amplicon sequencing and number of OTUs in *H*. *virescens* larvae. The bacterial communities of field larvae (F), laboratory larvae (L) and field-lab (AB/NoAB) larvae were characterized. The latter was reared in the laboratory for four generations after collection in the field. AB: treated with antibiotics (tetracycline); NoAB: not treated with antibiotics. Larvae of the field and laboratory strain were fed on three different plant species: cotton (C), chickpea (Ch), and tobacco (T). Field larvae: two sequencing pools per plant species; laboratory and field-lab larvae: one pool per plant species; # = number, Qual. seqs = quality filtered sequences.
(DOCX)

S3 Table Statistics of bacterial tag-encoded FLX amplicon sequencing and number of OTUs in individual *H*. *virescens* females. Females fed on three plant species as larvae: cotton (C), chickpea (Ch), tobacco (T); # = number, Qual. seqs = quality filtered sequences.
(DOCX)

S4 Table Statistics of bacterial tag-encoded FLX amplicon sequencing and number of OTUs in *H*. *virescens* eggs. Mothers of these eggs had fed on three different plant species as larvae: cotton (C), chickpea (Ch), tobacco (T); # = number, Qual. seqs = quality filtered sequences.
(DOCX)
