# Supplementary figures and images for: Variability of Bacterial Communities in the Moth Heliothis virescens Indicates Transient Association with the Host
Source: PLoS One. 2016 May 3;11(5):e0154514. doi: 10.1371/journal.pone.0154514 (PMC4854476; doi:10.1371/journal.pone.0154514)

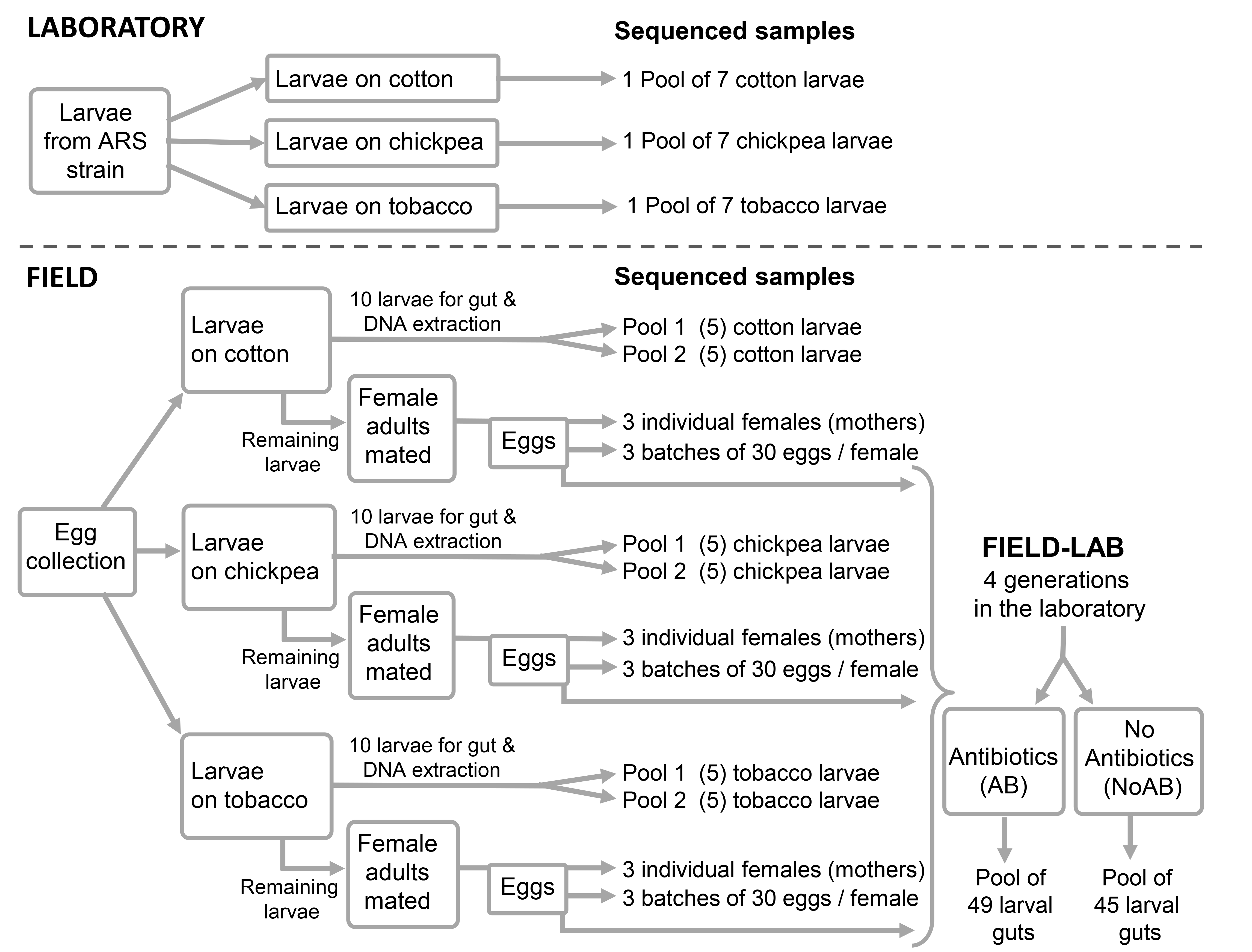

Supplement: S1 Fig — (TIF) [file pone.0154514.s001.tif]

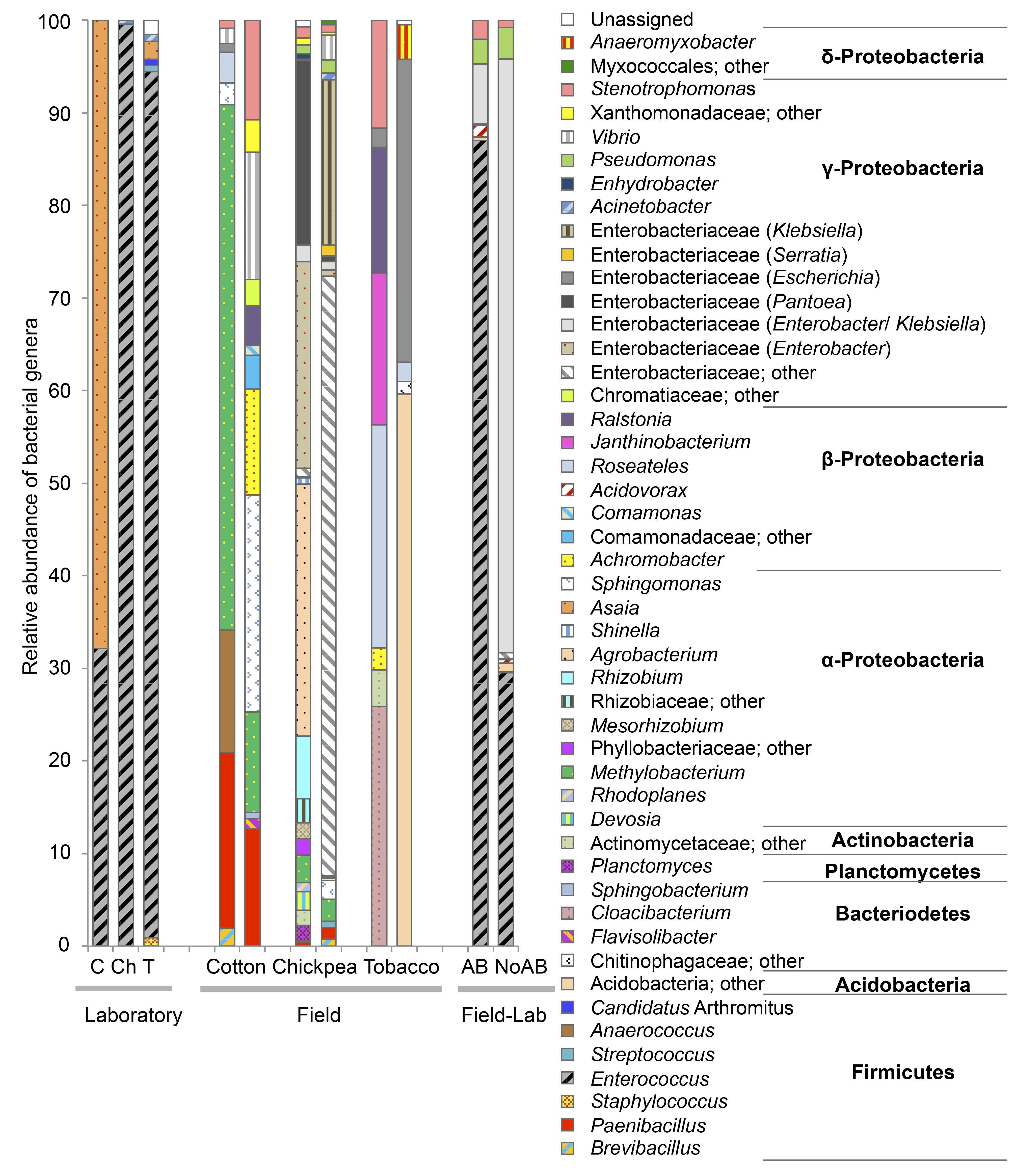

Supplement: S2 Fig — Rare OTUs that were not represented more than 0.5% in at least one sample are not included. Larvae originated from a laboratory colony, from the field or were collected from the field as eggs, after which they were reared in the laboratory for four generations (field-lab): AB: larvae received antibiotics treatment, NoAB: larvae received no antibiotics treatment. Field and laboratory larvae were grown on cotton (C), chickpea (Ch) or tobacco (T). Brackets around genera signify that there were bacterial candidates with > 97% identity, but support in the phylogenetic tree was weak [i.e. nodes that divide branches that contain different genera have bootstrap values equal to or below 10 (see Fig 1)]. (TIF) [file pone.0154514.s002.tif]

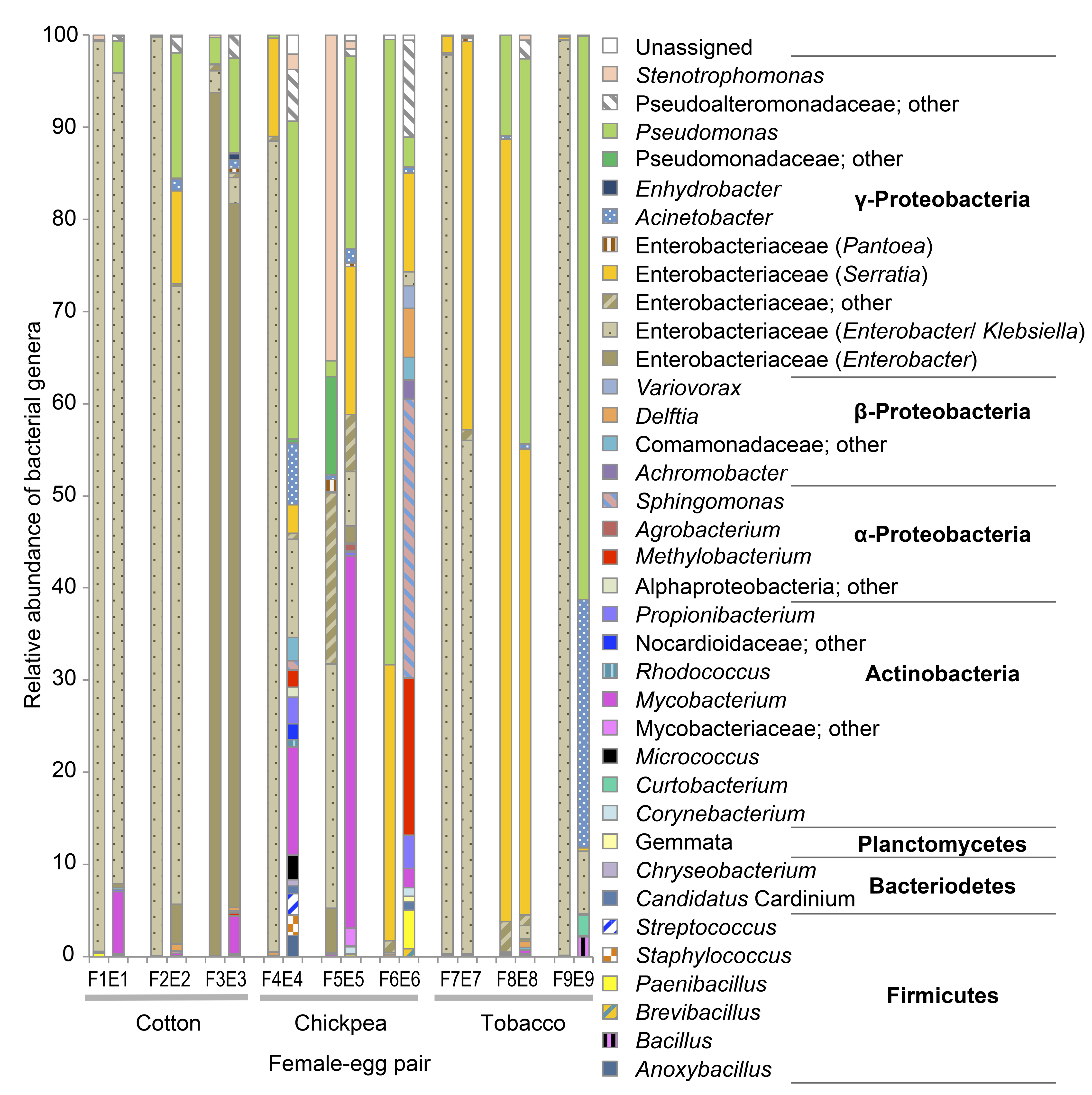

Supplement: S3 Fig — Females (F1-F9) and their eggs (E1-E9) are shown next to each other. Females had fed as larvae on three different plants: cotton (C), chickpea (Ch), tobacco (T). Rare OTUs that were not represented more than 0.5% in at least one sample are not included. Brackets around genera signify that there were bacterial candidates with > 97% identity, but support in the phylogenetic tree was weak [i.e. nodes that divide branches that contain different genera have bootstrap values equal to or below 10 (see Fig 1)]. (TIF) [file pone.0154514.s003.tif]
