# Supplementary material for: Variability of Bacterial Communities in the Moth Heliothis virescens Indicates Transient Association with the Host
Source: PLoS One. 2016 May 3;11(5):e0154514. doi: 10.1371/journal.pone.0154514 (PMC4854476; doi:10.1371/journal.pone.0154514)
Supplement: S1 Table — Laboratory larvae, larvae that were collected in the field as eggs (field), larvae that were collected in the field as eggs and were kept in the laboratory for four generations (field-lab), female adults and eggs. (DOCX) [file pone.0154514.s004.docx]

**S1 Table. Percentage of chloroplast and mitochondrial reads in the different sample groups.** Laboratory larvae, larvae that were collected in the field as eggs (field), larvae that were collected in the field as eggs and were kept in the laboratory for four generations (field-lab), female adults and eggs.

|  | **Laboratory larvae** | **Field larvae** | **Field-lab larvae** | **Female**  **adults** | **Eggs** |
| --- | --- | --- | --- | --- | --- |
| Chloroplasts | 1.51 | 21.72 | 0.06 | 0.0039 | 2.063 |
| Mitochondria | 0.19 | 4.83 | 0 | 0 | 0.078 |
