# Supplementary material for: Variability of Bacterial Communities in the Moth Heliothis virescens Indicates Transient Association with the Host
Source: PLoS One. 2016 May 3;11(5):e0154514. doi: 10.1371/journal.pone.0154514 (PMC4854476; doi:10.1371/journal.pone.0154514)
Supplement: S2 Table — The bacterial communities of field larvae (F), laboratory larvae (L) and field-lab (AB/NoAB) larvae were characterized. The latter was reared in the laboratory for four generations after collection in the field. AB: treated with antibiotics (tetracycline); NoAB: not treated with antibiotics. Larvae of the field and laboratory strain were fed on three different plant species: cotton (C), chickpea (Ch), and tobacco (T). Field larvae: two sequencing pools per plant species; laboratory and field-lab larvae: one pool per plant species; # = number, Qual. seqs = quality filtered sequences. (DOCX) [file pone.0154514.s005.docx]

**S2 Table. Statistics of bacterial tag-encoded FLX amplicon sequencing and number of OTUs in *H. virescens* larvae.** The bacterial communities of field larvae (F), laboratory larvae (L) and field-lab (AB/NoAB) larvae were characterized. The latter was reared in the laboratory for four generations after collection in the field. AB: treated with antibiotics (tetracycline); NoAB: not treated with antibiotics. Larvae of the field and laboratory strain were fed on three different plant species: cotton (C), chickpea (Ch), and tobacco (T). Field larvae: two sequencing pools per plant species (F1 and F2); laboratory and field-lab larvae: one pool per plant species; # = number, Qual. Seqs. = quality filtered sequences.

|  | **Laboratory larvae** | | |  | **Field larvae** | | | | | |  | **Field-lab larvae** | |
| --- | --- | --- | --- | --- | --- | --- | --- | --- | --- | --- | --- | --- | --- |
| **Sample** | **CL** | **ChL** | **TL** |  | **CF1** | **CF2** | **ChF1** | **ChF2** | **TF1** | **TF2** |  | **AB** | **NoAB** |
| # Reads | 30475 | 6917 | 5614 |  | 33880 | 2226 | 2590 | 2835 | 5356 | 3189 |  | 11464 | 10344 |
| # Qual. seqs | 30400 | 6750 | 5112 |  | 20963 | 1520 | 2318 | 2791 | 5333 | 3189 |  | 11443 | 10341 |
| # OTUs | 27 | 21 | 27 |  | 55 | 23 | 55 | 53 | 23 | 16 |  | 64 | 90 |
