# Supplementary material for: Variability of Bacterial Communities in the Moth Heliothis virescens Indicates Transient Association with the Host
Source: PLoS One. 2016 May 3;11(5):e0154514. doi: 10.1371/journal.pone.0154514 (PMC4854476; doi:10.1371/journal.pone.0154514)
Supplement: S3 Table — Females fed on three plant species as larvae: cotton (C), chickpea (Ch), tobacco (T); # = number, Qual. seqs = quality filtered sequences. (DOCX) [file pone.0154514.s006.docx]

**S3 Table. Statistics of bacterial tag-encoded FLX amplicon sequencing and number of OTUs in individual *H. virescens* females.** Females fed on three plant species as larvae: cotton (C), chickpea (Ch), tobacco (T); # = number, Qual. Seqs. = quality filtered sequences.

| **Sample** | **C-fem1** | **C-fem2** | **C-fem3** | **Ch-fem4** | **Ch-fem5** | **Ch-fem6** | **T-fem7** | **T-fem8** | **T-fem9** |
| --- | --- | --- | --- | --- | --- | --- | --- | --- | --- |
| # Reads | 7586 | 16398 | 7374 | 15853 | 13047 | 9509 | 13254 | 2895 | 16990 |
| # Qual. seqs. | 7586 | 16396 | 7373 | 15852 | 13047 | 9507 | 13156 | 2895 | 16990 |
| # OTUs | 33 | 44 | 34 | 65 | 72 | 65 | 54 | 38 | 57 |
