# Supplementary material for: Variability of Bacterial Communities in the Moth Heliothis virescens Indicates Transient Association with the Host
Source: PLoS One. 2016 May 3;11(5):e0154514. doi: 10.1371/journal.pone.0154514 (PMC4854476; doi:10.1371/journal.pone.0154514)
Supplement: S4 Table — Mothers of these eggs had fed on three different plant species as larvae: cotton (C), chickpea (Ch), tobacco (T); # = number, Qual. seqs = quality filtered sequences. (DOCX) [file pone.0154514.s007.docx]

**S4 Table. Statistics of bacterial tag-encoded FLX amplicon sequencing and number of OTUs in *H. virescens* eggs.** Mothers of these eggs had fed on three different plant species as larvae: cotton (C), chickpea (Ch), tobacco (T); # = number, Qual. Seqs. = quality filtered sequences.

| **Sample** | **C-egg1** | **C-egg2** | **C-egg3** | **Ch-egg4** | **Ch-egg5** | **Ch-egg6** | **T-egg7** | **T-egg8** | **T-egg9** |
| --- | --- | --- | --- | --- | --- | --- | --- | --- | --- |
| # Reads | 1346 | 18062 | 8461 | 1724 | 8033 | 466 | 27786 | 8890 | 19760 |
| # Qual. seqs. | 1241 | 17888 | 8384 | 503 | 7587 | 339 | 27784 | 8855 | 19746 |
| # OTUs | 37 | 108 | 81 | 48 | 134 | 30 | 82 | 70 | 124 |
